# The Everyday Moral Judge – Autobiographical Recollections of Moral Emotions

**DOI:** 10.1371/journal.pone.0167224

**Published:** 2016-12-15

**Authors:** André Körner, Nadine Tscharaktschiew, Rose Schindler, Katrin Schulz, Udo Rudolph

**Affiliations:** 1 Department of General Psychology and Biopsychology, Technische Universität Chemnitz, Chemnitz, Germany; 2 SRH Fachhochschule für Gesundheit Gera, Gera, Germany; University of Reading, UNITED KINGDOM

## Abstract

Moral emotions are typically elicited in everyday social interactions and regulate social behavior. Previous research in the field of attribution theory identified ought (the moral standard of a given situation or intended goal), goal-attainment (a goal can be attained vs. not attained) and effort (high vs. low effort expenditure) as cognitive antecedents of moral emotions. In contrast to earlier studies, mainly relying on thought experiments, we investigated autobiographical recollections of N = 312 participants by means of an online study. We analyzed a diverse range of moral emotions, i.e., admiration, anger, contempt, indignation, pride, respect, schadenfreude, and sympathy, by using a mixed-method approach. Qualitative and quantitative methods clearly corroborate the important role of ought, goal-attainment, and effort as eliciting conditions of moral emotions. Furthermore, we built categorical systems based on our participants’ descriptions of real-life situations, allowing for more fine-grained distinctions between seemingly similar moral emotions. We thus identify additional prerequisites explaining more subtle differences between moral emotion clusters as they emerge from our analyses (i.e., cluster 1: admiration, pride, and respect; cluster 2: anger, contempt, and indignation; cluster 3: schadenfreude and sympathy). Results are discussed in the light of attributional theories of moral emotions, and implications for future research are derived.

## Introduction

Imagine your daughter is awarded a scholarship for spending two years as a post-doc scholar at a highly prestigious university such as Harvard or Yale. Needless to say how proud you are. In contrast, imagine a good friend promised to help you with your removal, but fails to show up for some futile reason. Needless to say you feel angry. Interpersonal events like these, regardless whether they are positive or negative, are especially prone to experiencing moral emotions. Furthermore, these emotions often guide subsequent behavior. On a most general level, we might say that moral emotions like pride or anger, sympathy or schadenfreude, and the like, regulate our tendencies to approach versus avoid, to praise versus reprimand, and to reinforce versus correct. We also assume, and this characteristic shapes the methodology of the present paper, that these moral emotions evoke vivid memories of our interpersonal life, as they become an integral part of the recollections we tell when it comes to describing and explaining interpersonal events.

In the present paper, we investigate autobiographical recollections of moral emotions. We assume that these recollections will shed light on the eliciting conditions of our emotional life. More specifically, we focus on moral emotions which we experience vis-à-vis the actions of others, like anger, indignation, sympathy or schadenfreude. A common feature of these moral emotions is that all these require considerations of good and bad or wrong and right (for a summary, see [1]).

Our research is guided by three observations: First, the landscape of moral emotions includes many members, among them prototypes as well as close relatives to these prototypes [1]. Second, existing empirical evidence suggests that these moral emotions are similar in some, but different in other respects. Some of the differences are almost self-evident (as between anger versus gratitude), while others are quite subtle and more difficult to detect (as between anger and indignation). Third, the vast majority of research in the domain of moral emotions is characterized by two approaches: One approach is to confront participants with highly complex moral dilemmas, which are extraordinary and thus extremely untypical of our everyday interpersonal interactions. Another approach aims at systematic variation of contrasting antecedent conditions (as independent variables), and analyzes of the imagined effects of these variations on our emotional reactions. A potential disadvantage of the latter technique is that it might overestimate such isolated antecedent conditions. Our main goal is to find an alternative route to analyze our everyday moral-emotional responding in interpersonal actions. To do so, let us first briefly summarize the theoretical concepts that help us to better understand the multifold landscape of moral emotions.

### A Classification of Moral Emotions

Fritz Heider [2] proposed a classification of moral emotions by identifying their presumed cognitive antecedents (see also, [3]). According to this point of view, moral emotions are strongly determined by three concepts, that is, ought, effort, and goal-attainment. Considerations of ought (i.e., right and wrong), either vis-à-vis one’s own or other persons’ actions, include evaluations of whether a morally positive or negative goal is present. Furthermore, human actions may differ with respect to the amount of effort or intensity with which such goals are pursued. Finally, the respective goal, be it right or wrong from a moral perspective, can be attained or not attained. Ought, effort and goal-attainment explain large amounts of variance in moral emotions (for a summary, see [3]). Two other conceptual elements help to classify the variety of moral emotions, namely, (a) the target of the emotion, and (b) the evaluative or signal function the emotion serves.

Moral emotions can be classified according to their target: Emotions evaluating one’s own actions or characteristics, such as guilt, pride, regret or shame, have been referred to as self-directed emotions [4] or actor-emotions [3]. In contrast, emotions that are directed at other person’s actions or characteristics, such as admiration, anger, schadenfreude, scorn, or sympathy, have been labeled as other-directed emotions [4] or observer emotions [3].

Moreover, all moral emotions contain an evaluative function: That is, positive moral emotions are elicited following one’s own (actor emotions) or another person’s (observer emotions) morally positive behavior, i.e., actions meeting or exceeding positive moral standards like help-giving to someone in need or investing effort to attain a morally positive goal. In contrast, negative moral emotions occur after one’s own (actor emotions) or other’s (observer emotions) morally negative behavior, i.e., transgressions of moral standards like lying or cheating or not investing effort to attain a positive goal [1,5–7].

In sum, we conclude that positive moral emotions are elicited when morally positive goals are pursued, especially when effort is invested to attain these goals, whereas negative moral emotions occur given that either negative moral goals are pursued, or effort is not invested to attain morally positive goals (thus violating a ‘norm of effort’).

### The Functional Value of Moral Emotions

In the present article, we examine the most important and prototypical moral observer emotions, that is, admiration, pride, respect, and sympathy as positive moral observer emotions, and anger, indignation, contempt, and schadenfreude as negative moral observer emotions. For sake of simplicity, we do not include synonyms, or emotions referring to only restricted and specific contexts are not addressed. The cognitive antecedents of these eight emotions will now be considered briefly.

### Positive Observer Emotions

Admiration, pride, and respect represent positive signals to those who are the targets of these emotions, conveying a positive reinforcement of the underlying behavior that elicited the emotion [8]. Thus, admiration, pride, and respect are typically experienced vis-à-vis morally positive behaviors, that is when moral standards are met or exceeded. Examples are successes due to high effort, extraordinary talents and skills, or especially praiseworthy behavior [3,4,7,9–18].

#### Admiration

Admiration has been characterized as an ‘other-praising’ or ‘approval-emotion’ elicited by especially praiseworthy behaviors of others or observing others exceptional skills and talents [9,19]. According to Algoe and Haidt [9], admiration motivates an observing person to work harder to achieve her/his own goal.

#### Pride

Pride may emerge as a positive evaluation of either a person’s ability or other stable characteristics (alpha-pride), or can be based on evaluations of behaviors and especially a person’s invested effort (beta-pride) [20].

#### Respect

Respect has high similarities to both admiration [1,9] and observer-pride [3]: It occurs after morally positive behavior (e.g., investing effort to attain morally positive goals) and/or having high ability [3].

### Negative Moral Emotions

We suggest that negative moral emotions represent negative signals to those who are the target of these emotions. The expression of an emotion like anger represents a stop-signal, signaling that the anger-eliciting behavior or characteristic of the interaction partner is not desirable [1]. Previous analyses (e.g. [1,4]) revealed that our affective lexicon is even more nuanced when it comes to such negative signals.

#### Anger

While the causes of anger can be either impersonal or personal (see, [2]), we restrict ourselves to the interpersonal expression of anger. Weiner [4] characterizes anger as an effort-related emotion, arising when another person is held as responsible for a transgression or failure (he/she “could and should have done otherwise” [11,15]).

#### Indignation

Indignation has also links to responsibility [4]. However, as compared to *anger*, people need not be personally involved in the emotion-eliciting situation, i.e., people may feel indignation especially in situations involving another person doing harm to a third person [21].

#### Contempt

According to Weiner [4], contempt is an ability-related emotion. In a similar vein, Hutcherson and Gross [22] state that contempt is related to perceived incompetence. Furthermore, contempt is experienced for violations of community, that is, e.g., an individual’s obligations within a society [23].

### Discordant Emotions

Discordant Emotions differ with respect to their hedonic and functional qualities [2]. This is the case for sympathy and schadenfreude. For sympathy, it feels sad to experience this emotion, while it sends a positive signal to the person in need, increasing the likelihood that help will be provided (e.g. [3]). In contrast, schadenfreude feels joyful (as someone takes joy in the misfortune of others), at the same time, it feels very bad to be the target of schadenfreude [1]. Although these two are very complex emotion, children can feel and display schadenfreude and sympathy already at an age of about three to four years [24,25]. Sympathy is linked to prosocial actions [4] (for a summary, see [26]), whereas schadenfreude predicts absence of help-giving [24,25].

### Empirical Approaches to Moral Emotions

The vast majority of empirical studies on moral emotions has been based on thought experiments, thus asking participants to imagine different kinds of emotion-eliciting situations, and to indicate the degree of different moral emotions they would feel in the position of a certain person described in the respective scenario. This approach misses to deeply analyze the causal antecedents as it disregards real-life experiences for prototypical moral emotions. Herein we will study self-reports in the tradition of phenomenological approaches [10,27].

### Aims and Expectations

We provide a test of a conceptual analysis of moral emotions by means of a methodology that has not yet been applied in the present context. That is, in contrast to previous thought experiments, we analyze actual personal experiences recalled from autobiographic memories. We therefore employ a mixed method approach (combining quantitative and qualitative methods) to analyze autobiographical recollections of real life experiences of *admiration*, *pride*, *respect*, *sympathy*, *anger*, *indignation*, *contempt*, and *schadenfreude*. We use concepts of ought (O), goal-attainment (GA), and effort (E) [28] as predefined coding categories for a deductive content analyses [28].

Among the numerous approaches to explain moral emotions, we chose Heider’s naïve action analysis [2] as it is a comprehensive approach into which other, more specific theories can be integrated. That is, as has been illustrated above, the predominant approach to moral emotions is to analyze a limited set of moral emotions and some of their eliciting conditions. A highly valuable example of this kind of approach are the specific conditions that are important to our understanding of a subset of moral emotions, the so-called self-conscious emotions (i.e., shame, guilt, embarrassment, and pride, see e.g., [20,29]) or other-condemning emotions (anger, indignation, disgust, [3,4,7,9–18]). In a similar vein, several appraisal-related approaches to moral emotions identified several antecedent appraisals (e.g., novelty, agency, valence, pleasantness, interestingness, compatibility with standards, legitimacy) important to specific (moral) emotions [30,31] (for a detailed overview, see [32]).

Two features of Heider’s naïve action analysis are especially important when it comes to moving toward a comprehensive theory of moral emotions: First, Heider’s approach aims at an understanding of our common-sense intuitions of interpersonal relations, and therefore, of course, aims to explains our moral intuitions and feelings as well. Second, Heider [2] identified a couple of concepts which he regarded as universal elements of social interactions. Most prominent among these are the concepts of ought, goal-attainment, and effort (as viewed from a lay-person’s perspective). As a consequence, Heider’s concepts represent an adequate starting point to explain a large range of moral emotions, which is the aim of our study.

In addition to Heider’s seminal work in this field, we are also interested in emotion-specific contextual factors that determine our moral-emotional responding and go beyond the latter categories. Therefore, we form additional coding categories by inductive coding strategies that refer to grounded theory in the line of Glaser and Strauss [33]. In doing so, we (1) analyze the impact of ought, goal-attainment, and effort as antecedent conditions of moral observer-emotions, (2) extend the number of antecedent conditions for moral emotions investigated by means of autobiographical experiences, and (3) provide a more precise and more comprehensive analysis to discover the subtlety of admiration, pride, respect, sympathy, anger, contempt, indignation, and schadenfreude.

#### Positive moral observer emotions

We expect that the majority of the experiences of *admiration*, *pride*, and *respect* contain situations in which morally positive goals are attained due to high effort (O+, GA+, and E+).

#### Negative moral observer emotions

The majority of the descriptions of the experience of *anger*, *indignation* and *contempt* should involve situations in which morally negative goals are attained (O–, GA+), especially when effort had been invested (O–, GA+, E+). Additionally, for *anger* we also expect eliciting situations where people fail to attain positive goals due to a lack of effort.

#### Discordant emotions

*Sympathy* should predominantly occur when another person fails to attain a morally positive goal. This should be even more the case when much effort is put it to attain this positive goal, but this nevertheless fails (O+, GA–, E+). Furthermore, *sympathy* should also be elicited when effort is unmentioned. For *schadenfreude* also a negative goal-attainment should be a crucial prerequisite. In contrast to sympathy schadenfreude should be much more likely elicited in situations where a person pursues a morally negative goal (O–, GA–). Again, this pattern should apply when high effort is put in or is not mentioned at all.

## Method

### Ethical Aspects

We did not use any kind of deception. The study entirely conforms to the ethical guidelines of the APA (http://www.apa.org/ethics/code/). Within the present online study, our participants were fully informed about the study by presenting an information page right at the beginning of the survey. This information provided detailed information about the goals of the study, the general procedure, and the expected time frame for participating in this study. Also, participants were informed that it is possible to cancel their participation at any time. We also pointed out that participation was fully anonymous, and no personal data would be processed. In addition, participants received contact details in case any questions or problem might arise. By clicking a button, the participants agreed to participate and thereby gave their informed consent. At the end of the study, participants again had the opportunity to either submit their data or to cancel their participation without any disadvantages or consequences. We also had a health care professional for urgent cases as a backup to support the participants in any case of emotional disturbance. The Ethics Committee of the Technische Universität Chemnitz approved the study presented here in a letter of inquiry.

### Mixed Methods Approach

Mixed methods approaches are a vital pathway for gaining a deeper insight into a person’s subjective understanding of events. Thus, studies combining quantitative and qualitative research are a growing field [34], used in many areas as for example counseling, nursing, and educational psychology (see also, [35]). In our view, the addition of qualitative methods is especially promising in emotion research, as we deal with subjective perceptions within a constant stream of interpersonal interactions. Mixes methods help to systematically build theories, rather than just testing a specific assumption. This offers a deeper understanding of the links between theory and empirical findings, help to question theoretical assumptions, and start developing new theories [36]. Up to date qualitative approaches lead a miserable existence in psychology [37] where only 1% of suitable APA publications accounted for such methods [38]. Fortunately, more and more mixed method research designs evolve to obviate methodical quarrels [34].

One argument often used against qualitative analyses is that they might lack reliability and validity. Furthermore there is often disunity even among qualitative researchers about methodical distinction and techniques [28]. A solution presented by Denzin [39] calls up researchers to explicitly state the type of technique they used, make clear how they obtained the results, and use triangulation methods. So far, guidelines have been developed for a wide branch of quantitative research, like QUOROM [40], MOOSE [41], STROBE [42], STARD [43], or CONSORT [44]. Alas, a formal standard for reporting qualitative has been missing for a long time. Fortunately, broadly accepted guidelines as to how report qualitative research has been developed over the past decade [COREQ, 45]. We rely to these standards by giving precise information concerning our participants, the research team, aspects of reflexivity, the study design, and analytic strategies.

### Participants

A total of *N* = 389 participants completed an automated online interview that had been announced through several notice boards and mailing lists at the Technische Universität Chemnitz and other German universities. Students in Chemnitz received partial course credit for participation. We added an option at the beginning of the questionnaire for participants to decide whether they wanted to take part “in real” or to just “visit” the questionnaire (which was used by n = 18 persons). This method can reliably detect real drop-outs [46]. Non-finished, visiting, and futile questionnaires (implausible processing times) were disregarded from further analysis. The real dropout-rate for the online test was 78 out of 389 (20.1%); hence, the final sample consisted of *N* = 312 participants. The average time to complete the questionnaire for these participants was *M* = 14.31 min (*SD* = 3.48). Students received partial course credits for participation.

About 91% (*n* = 283) of the participants were students of the Technische Universität Chemnitz. Other participants came from all over Germany. The majority, 85% (*n* = 265) of the participants, were female. Their age ranged from 15 to 60 years with a mean age of *M* = 23.2 years (*SD* = 6.69). None of the participants had a relation to the researchers, nor were they informed about the specific research ideas.

### Research Team and Reflexivity

The research team consisted of five researchers, three of them female (Nadine Tscharaktschiew, Katrin Schulz, and Rose Schindler), and two of them male (Udo Rudolph and André Körner). Furthermore, we trained two female student assistants in qualitative research and coding procedures. All five researchers obtained a German degree in psychology and have been working at the Department of Psychology in Chemnitz at the time of data collection and data analyses. All of them specialized in moral emotion research. Furthermore, as a research team, we investigate specific moral emotions in different contexts and focus on the development of moral emotions in childhood. We also investigate physiological correlates of moral emotions. The sampling process as well as the participants’ allocation to different conditions of the questionnaire were anonymous. As all researchers had been lecturers at the university, the participants might have known them from courses or lectures they attended. However, none of the researchers knew who was participating in the study, as data collection was completely anonymous. Thus, there was no relevant relationship between researchers and participants.

### Data Collection

The participants in the online interview were randomly assigned to one of eight emotion conditions (admiration, pride, respect, sympathy, anger, indignation, contempt, and schadenfreude). After a brief collection of demographic data, they received a short preview and an introduction to the interview. Participants were asked to recall an event from their past when they had experienced the specific emotion as an observer (‘We are interested in incidents and situations in which you had feelings towards another person–as an observer.’) We highlighted that there was no time frame for this recalled event (be it weeks, months, or years). Rather, our criterion was that the selected past event could be recalled easily, and that respondents were willing to reveal this event.

**Fig 1:**
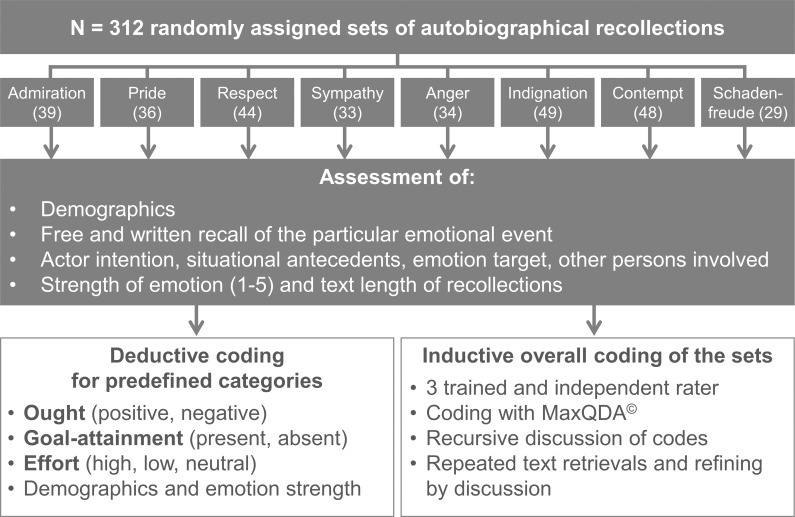
Research strategy and mixed-method approach at a glance. The participants were allocated randomly to one of the eight emotions and interviewed online by specific aspects of an emotional event of their own past. The resulting text files then were analyzed using deductive and inductive coding.

We structured the recall process by asking for specific antecedents of the event. Participants then wrote their answers into a free text field, without having any time or space restrictions. The questions were as follows, (1) ‘What exactly elicited the (emotion)? What happened in detail? Please describe the event as precisely as you can.’ After producing a free text, we asked the participants (2) ‘What did the other person do or wanted to do?’ Then we returned to the antecedents of the event by asking for the other person’s actions: (3) ‘What happened in the situation beforehand? What did the other person do?’ Furthermore, we were interested in the context of the situation: (4) ‘Who else was involved in the situation and what is your relation to those persons: are these friends, acquaintances, partners, relatives, colleagues…’ Finally, participants rated the intensity of their emotion on a 5-point unipolar scale (strength of emotion, 1 = “slight” to 5 = “very strong. Fig 1 gives an overview of data acquisition and data analyses. The questions and resulting answers of the participants’ online interviews were merged into an rtf-formatted document and imported into a computer program (MaxQDA) for qualitative analyses [47,48]. The resulting files are available from an Open Science Framework (https://osf.io/tjbex/?view_only=aa8eee6502b14cf4bedfda3ed8649d4c).

### Data Analyses

Within a mixed-method approach, we used a triangulation process consisting of both quantitative and qualitative research strategies, including deductive and inductive coding. That is, all interviews were coded by at least two researchers [see, 39,49]. Furthermore, we used common techniques of qualitative research: (1) memos for category building and documenting each step of the research process, (2) several text retrievals for all eight emotions, and (3) jointly discussing the evolving categories.

In a first step, we analyzed the concepts ought, goal-attainment, and effort, based on deductive coding of written recalls. In line with proposals by Mayring [28], we used qualitative content analyses, and therefore set predefined categories for the concepts of ought, goal-attainment, and effort within the autobiographical recalls. Minimal definitions were as follows: (1) Ought: the described action of the observer is morally positive (O+) or negative (O–). (2) Goal-attainment: the goal of the actor was attained (GA+) or not attained (GA–). (3) Effort: The actor invested high effort (E+), he did not so (E–), or effort was unclear (neutral) within the written recalls (E(n)). We identified prototypical text passages for the three categories to use these as anchors and to establish coding rules. Two coders coded the interviews by means of these predefined categories at hand. We built memos for ambiguous texts and resolved all coding problems by discussion.

In a second step, we used inductive coding techniques according to grounded theory [33], to better understand the specific features of otherwise similar emotions. Thus, we were able to build a category system in a stepwise fashion, without a-priori assumptions. We trained two female student assistants, who individually coded the interviews, with respect to these coding procedures. We then discussed the emerging categories within the entire research team. This technique of constant comparison also allows for a discovery of discrepancies within the coding procedure.

## Results

### Strength of Emotions and Length of Interviews

**Fig 2:**
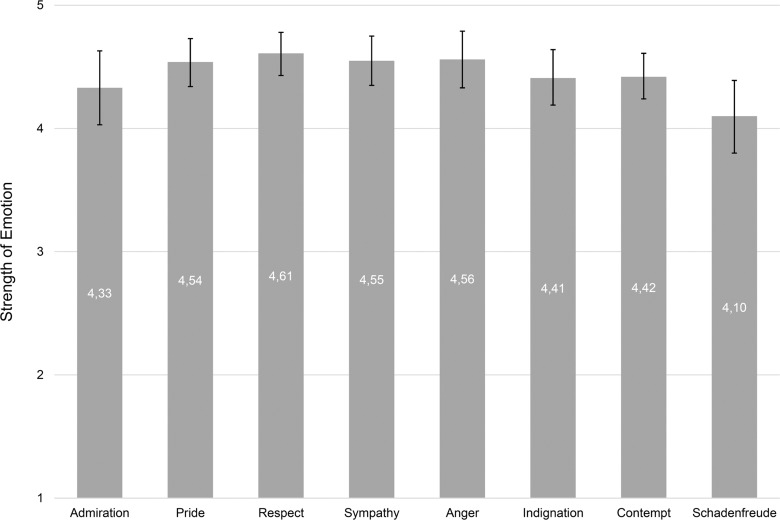
Mean strength of emotional reactions for the recalled events of the eight different emotions. The bars show the means for the strength of the participants’ emotional experience within the autobiographical events (5-point scale from 1 = weak to 5 = very strong). Areas around mean values indicate 95% confidence intervals of 10,000 bootstrapping trials.

The emotion events recalled by the interviewees did not differ in terms of emotional strength of the autobiographical event. A Levene test revealed that the variances of the emotional strengths of all eight emotions were homogeneous, *F*(7, 304) = 1.105, *p* = .36. All interviews were experienced as highly emotional. Overall emotional strength was *M* = 4.45 (*SD* = 0.71), ranging from *M* = 4.10 (*SD* = 0.77) for schadenfreude to *M* = 4.61 (*SD* = 0.58) for respect. Fig 2 shows the exact means for all eight emotions.

A bootstrapping procedure (10,000 trials) revealed a confidence interval of CI95% = (4.37; 4.53) for the overall emotional strength. When comparing the means of emotional strength by a univariate ANOVA, we found no differences between the eight emotions, *F*(7, 304) = 1.846, *p* = .08, *n*^2^ = .02. A post-hoc test also did not find homogeneous subgroups when using Scheffé procedure, *p* = .19. At least the Tukey-B post-hoc test built two homogenous subgroups (*p* < .05), by including schadenfreude and excluding respect in group 1 and vice versa for subgroup 2.

**Fig 3:**
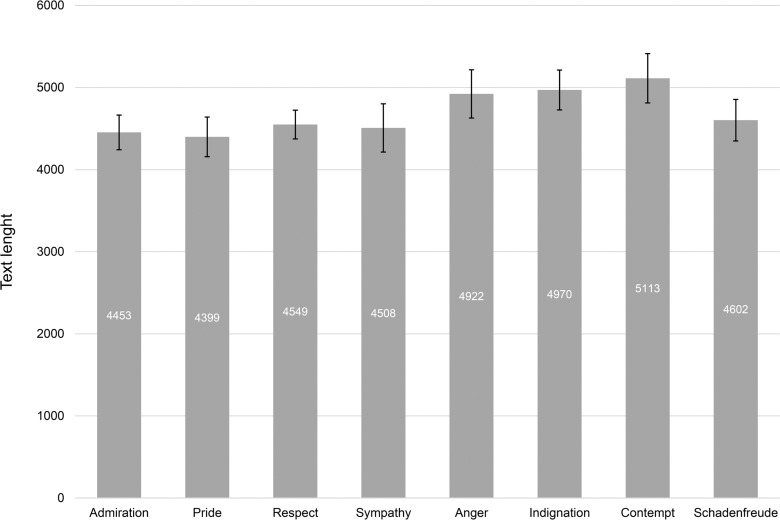
Length of text files for the interviews of the eight different emotions. The bars show the means for the text length of the participants’ autobiographical events expressed by bytes of the text files. Areas around mean values indicate 95% confidence intervals.

We also analyzed the length of the written recollections. Fig 3 shows the file size of the texts measured as number of bytes. The variances of the text length for the eight emotions were not homogeneous, *F*(7, 304) = 2.543, *p* = .02. Overall text length for the interviews was *M* = 4,715 bytes (*SD* = 825), ranging from *M* = 5,113 (*SD* = 1,035) for contempt to *M* = 4,399 (*SD* = 703) for pride. A bootstrapping procedure (10,000 trials) revealed a confidence interval of CI95% = (4,623; 4,807) for the overall text length. When comparing the means of text length by a univariate ANOVA, we found a significant difference between the eight emotions, *F*(7, 304) = 4.887, *p* < .01, which indicated a medium effect (*n*^2^ = .10). Respondents wrote longer texts for negative emotions (anger, indignation, and contempt).

A Scheffé post-hoc test identified homogeneous subgroups: The first subgroup excluded the negative emotion contempt, but included the positive emotion pride. Hence, this aggregation was not a significant finding, with *p* = .20 for the first subgroup and *p* = .07 for the second subgroup. However, the Tukey-B post-hoc test also built three homogenous subgroups, which was a significant finding with *p* < .05. A first subgroup consisted of three positive emotions (admiration, pride, and respect) and both discordant emotions (schadenfreude, sympathy), as well as the negative emotion anger. A second subgroup excluded pride and included indignation from the prior subgroup. The third and last subgroup consisted of schadenfreude together with the three negative emotions (anger, indignation, and contempt).

### Deductive Analyses of Ought, Goal-attainment, and Effort

As can be seen Table 1 the concepts of O, GA, and E have been used extensively by our participants when describing their personal experiences. O and GA were addressed within all autobiographical recollections; effort was addressed in the vast majority of cases 64.4%). Table 2 shows the two most frequent patterns of O, GA, and E as they emerged from the descriptions of the participants. We will describe these patterns in more detail for each of the respective emotions below. A complete overview of all patterns extracted from our subjects’ descriptions can be found in the dataset we provide in the Open Science Framework.

**Table 1:** Frequencies of Predefined Coding Categories in the Content Analyses.

Observer Emotion (N)	Ought			Goal-attainment			Effort		
	(+)	(–)	(n)	(+)	(–)	(n)	(+)	(–)	(n)
Admiration (39)	38	1	0	39	0	0	26	2	11
Pride (36)	36	0	0	36	0	0	31	0	5
Respect (44)	44	0	0	44	0	0	34	0	10
Sympathy (33)	33	0	0	0	33	0	13	3	17
Anger (34)	8	26	0	25	9	0	12	11	11
Indignation (49)	7	42	0	36	13	0	12	9	28
Contempt (48)	3	45	0	44	4	0	23	6	19
Schadenfreude (29)	19	10	0	1	28	0	11	8	10

*Notes*. O = Ought (moral standard), with (+) for morally positive and (–) for morally negative. GA = Goal-attainment with (+) for present and (–) for absent. E = Effort with (+) for high effort, (–) for low effort, and (n) for unclear.

**Table 2:** Frequencies for Patterns of Ought, Goal-attainment, and Effort Within the Eight Emotions.

Emotion (N)	Main pattern	N_1_ (%)	Second pattern	N_2_ (%)	N_cum_	%covered
Admiration (39)	O+ GA+ E+	25 (64.1)	O+ GA+ E(n)	11 (28.2)	36	92.3
Pride (36)	O+ GA+ E+	31 (86.1)	O+ GA+ E(n)	5 (13.9)	36	100.0
Respect (44)	O+ GA+ E+	34 (77.3)	O+ GA+ E(n)	10 (22.7)	44	100.0
Sympathy (33)	O+ GA–E(n)	17 (51.5)	O+ GA–E+	13 (39.4)	30	90.9
Anger (34)	O–GA+ E+	11 (32.4)	O–GA+ E(n)	11 (32.4)	22	64.8
Indignation (49)	O–GA+ E(n)	26 (53.1)	O–GA–E(n)	8 (16.3)	34	69.4
Contempt (48)	O–GA+ E+	22 (64.1)	O–GA+ E(n)	19 (39.6)	41	85.4
Schadenfreude (29)	O+ GA–E–	8 (27.6)	O–GA–E(n)	8 (27.6)	24	82.8
	O+ GA–E(n)	8 (27.6)				

*Notes*. O = Ought (moral standard), with (+) for morally positive and (–) for morally negative. GA = Goal-attainment with (+) for present and (–) for absent. E = Effort with (+) for high effort, (–) for low effort, and (n) for unclear. Main pattern presents the most frequent combinations of O/GA/E found for the specific emotions with frequencies (N_1_) in relation to all interviews (%). Second pattern presents the second most frequent combinations of O/GA/E found for the specific emotions with frequencies in relation to all interviews (N_2_, %). N_cum_ = Total number of interviews covered by N_1_ and N_2_. “%covered” = N_cum_ in relation to all interviews.

#### Positive moral emotions

As expected, the three positive moral observer emotions admiration, pride and respect typically occurred when morally positive goals were attained and effort had been invested to attain the respective goal (O+, GA, +E+, see Table 2). Furthermore, these emotions were also elicited when no specific information about effort was present (O+ GA+ E(n)). Overall, more than 90% of all text passages for positive moral observer emotions were covered by these two patterns.

#### Negative moral emotions

The behavior described for the negative emotions anger, indignation, and contempt was morally wrong in most of the recollections, although this was not as clear as we found it for the positive emotions (see Table 1). This was due to the fact that goal-attainment seemed to play a minor role for negative emotions, as the recollections refer to both attainments and non-attainments of a given goal. Besides contempt, for a large number of recollections effort was not mentioned at all. This resulted in a higher diversity of recollections of negative emotions (see Table 2). Regardless of effort, anger and indignation were experienced predominantly when negative goals were attained (O–, GA+), whereas the next frequent pattern for contempt was O–, GA–. For anger (23.5%) and indignation (14.3%). We also found recollections describing a morally positive goal that is not attained due to lack of effort (O+, GA–, and E–).

#### Discordant moral emotions

The two discordant emotions sympathy and schadenfreude typically occurred when a goal was not attained. For schadenfreude, there are two main patterns: Schadenfreude is mainly elicited (55.2%) when a morally positive goal is not attained (O+ GA–), or when a morally negative goal is not attained (O–GA–; 27.6%). In regard to the first more frequently pattern, schadenfreude was either elicited when effort hasn’t been invested to attain the morally positive goal (27.6%) or when no specific information about effort was present (27.6%). In contrast, more than 90.9% of all text passages for Sympathy included morally positive goals which were not attained (O+ GA–). Furthermore, sympathy was either elicited when effort has been invested to attain the respective goal (51.5%) or when no specific information about effort was present (39.4%).

### Inductive Analyses of Emotion-Specific Contextual Factors

As noted earlier, qualitative methods are more than to allocate given descriptions to empirical relatives. Thus, our aim is to identify additional specifics of the emotions investigated here. We will do so by giving summaries for each of the eight observer emotions. Furthermore, we want to shed light on the similarities and differences of the given emotions. For each emotion we quote one concise depiction from our participants’ recollections. For a more comprehensive list of quotes, the reader is referred to Appendices A-C.

#### Positive moral observer emotions

In line with our expectations and without exception, all three positive moral emotions were elicited by morally positive behaviors (e.g., specific performances or achievements, coping with critical life events, help-giving and other humanly behaviors) or morally positive traits (e.g., aptitude or personality), and were often accompanied by diverse positive feelings (e.g., being impressed or fascinated or experiencing happiness). We will now consider more fine-grained differences between these three positive moral emotions as reported in the respective autobiographical recollections. In this regard, we will refer to major findings of our inductive analyses (see in Table 3) and will illustrate these findings by quoting typical recollections from our participants.

**Table 3:** System of Categories and their Frequencies for Respect, Admiration, and Pride.

Category	Pride	Admiration	Respect
Subcategory	(N = 36)	(N = 39)	(N = 44)
**C loseness of relation to the actor**			
Close	35	24	28
being involved in the situation	21	–	–
Distant	1	10	10
Unfamiliar	–	5	4
**Actor personality**			
Likeable	–	–	4
Unlikeable	–	–	1
**Personality development**	17	–	–
**Higher hierarchical position**	–	–	4
**Expressed additional feelings**			
being glad	2	–	–
joy/ happiness/ luck	3	–	–
being impressed or overwhelmed	2	2	9
astonishment	–	1	1
fascination	–	4	1
admiration	–	–	4
pride	–	–	3
enthusiasm	–	–	1
awe	–	–	1
**Specific competence of the actor as a person**			
ability/ competence	6	10	3
personality/ traits	3	18	8
self-discipline	–	6	–
maturity	–	–	1
humanity	–	6	6
optimism/ vitality	–	–	2
mindset / will power	–	1	2
selflessness/ altruism	1	–	12
**The actor is mastering a specific situation**			
coping with critical life event(s)	3	10	6
related to sth. unexpected	–	3	–
accepting sth. negative or reaching a higher goal	–	13	–
to overcome opposition(s)	11	6	3
proceed to new/ strange situations	2	–	5
**Performance/ behavior**			
commitment	–	–	6
readiness to help	1	–	8
multiple workload/ burden?	–	6	–
context of achievement/ performance	26	9	16
**Miscellaneous**			
observer is not able to show the behavior/ ability her/ himself	–	14	15
self-motivation / role model function	3	3	6
earn admiration	–	3	–
similar situation / hobby	–	19	–
prior hesitation	6	–	–

*Notes*. Numbers indicate the recollections containing the described category/ subcategory. Dashes indicate that none of the recollections have such a category/ subcategory. The numbers in bracket beneath pride, admiration, and respect show the number of recollections for these emotions.

As was also expected, the concepts of ability/competence (pride: 6 of 36 recollections; admiration: 10 of 39; respect: 3 of 44) or personality/traits (both referring to aspects of a persons’ self) were also covered for all three emotions (pride: 3 of 36 recollections; admiration: 18 of 39; respect: 8 of 44). However, we also discovered a difference with regard to the positive perception of other person’s abilities or traits for pride on the one hand, and admiration and respect on the other. Let us analyze this in more detail.

Pride. As expected, in contrast to admiration and respect, pride was almost exclusively experienced within close relationships (35 of 36 recollections). That is, pride was usually directed at family members, partners or good friends. Furthermore, this emotion was very often experienced with regard to good performances in achievement contexts (26 of 36 recollections), for example:

“My cousin (…) played very well. He had to play in front of many people for the first time, approximately 100, and he was very nervous before performing.” (Subject 21)

Additionally, pride was also experienced when the observer is involved her/himself (21 of 36 recollections) and was often elicited in contexts referring to personality development (17 of 36 recollections). Returning to the difference in the perception of others positive abilities or traits, interestingly, pride was typically related to ability or achievements the observing person does possess her/himself, as none of the 36 recollections mentioned otherwise. In contrast, the reverse is true for admiration (14 of 39 recollections) and respect (15 of 44 recollections; see below), as these were experienced vis-à-vis features or characteristics we do not possess ourselves.

Admiration. As just described, admiration was often experienced with respect to another person’s achievements or abilities, given that the observing person did not possess these achievements or abilities. For example, here is a person participating in a dance class who admired the observed person for her excellent dancing skills. This example also shows that admiration often occurred with respect to characteristics which are important to the observer:

“It was the first time that we watched the competition ballroom dance training in our current dance sports club. In doing so I noticed a dancer–younger than me–she moved wonderfully and danced with great routine. Her whole posture and her expression triggered great admiration (in me), even more so because at that time I just had have a beginner's course for dancing.” (Subject 44)

As compared to pride, admiration was less often experienced in achievement contexts (9 of 39 recollections, compared to 26 of 36 for pride, and 16 of 44 for respect) and more often directed at personality and/or ability characteristics of the admired person. Moreover, admiration was also elicited in situations in which the observed person accepted certain negative aspects of events or brings sacrifices to achieve a higher goal (13 of 39 recollections).

Respect. To some extent, respect was also related to achievement and performance (16 of 44 recollections, see subject 30 below), which was less often found for admiration (only 9 of 39 recollections). However, in contrast to pride, respect was not (almost) exclusively reported for close relationships, but also related to more distant persons (pride: 1 of 36 recollections, respect: 10 of 44 recollections).

“The new trainer managed to attract everybody and brought them to push themselves to their personal limits. He did this with a high level of assertiveness and seriousness. I felt respect towards the coach. Until then, he was just a casual acquaintance.” (Subject 30)

Among the three positive moral observer emotions, respect was the emotion most often accompanied by being impressed by the observed person (9 of 44 recollections). Furthermore, respect was often related to altruism and help-giving (12 of 44 recollections). Moreover–as already seen for admiration–respect often occurred with regard to abilities or competencies the observer was not able to show or not have her/himself (15 of 44 recollections). Additionally, respect–as is also the case for pride and respect–was generally related to aspects of the observing person’s personality and abilities.

#### Negative observer emotions

As for the positive emotions, Table 4 shows the inductive coding for the three negative emotions (anger, indignation, and contempt) into a comprehensive categorical system. One common feature for these negative emotions was, that they are accompanied by a large number of additional feelings and fine graded emotions and perceptions, most of them with a negative hedonic quality. We will now describe specific characteristics of these emotions by prototypical quotes from the interviews.

**Table 4:** System of Categories and their Frequencies for Anger, Indignation, and Contempt.

Category	Anger	Indignation	Contempt
Subcategory	(N = 34)	(N = 49)	(N = 48)
**Closeness of relation between actor and observer**			
Close	23	22	9
barely known	8	15	19
stranger	3	12	20
**Person affected by the actors “wrong” behavior**			
observer himself	34	35	18
other persons	–	12	25
animals	–	2	–
**Blocking (the observer)**			
of fulfilling a given task	4	6	–
of control over privacy	2	–	–
in terms of hierarchy	2	4	–
in reaching an own goal	6	–	–
of a conflicts solution	4	–	–
of social relations between actor-observer	8	4	–
of social relations from observer to 3^rd^ person	4	–	–
**Relation of the observer to a 3**^**rd**^ **party affected by the actor’s wrong behavior**			
close	–	–	14
barely known	–	–	4
stranger	–	–	7
**Experienced inequity of the actor**	6	–	11
**Disappointment of expectations**	29	–	–
**Threat for self-esteem**	17	–	–
**Explicit focus on a norm or standard**	–	20	–
**Behavior of the actor seen as**			
incomprehensible	–	9	–
unreasonably	–	20	–
exaggerated	–	7	12
extortive	–	–	1
breaking a personal taboo	–	–	14
disrespectful	–	–	16
dishonest	–	–	10
**Further experienced feelings and perceptions**			
defenselessness	3	–	–
anger about oneself	3	5	–
anger about the observer	–	–	5
indignation	–	–	3
rage	9	5	8
frustration	2	4	6
shame	4	1	–
embarrassment	–	–	2
offendedness	5	–	–
to feel like left alone	2	–	–
betrayal	2	–	–
guilt	1	–	–
inequity	4	–	–
breach of confidence	2	–	–
consternation	1	–	3
sadness	–	2	–
disgust	–	1	–
shocked	–	5	4
fatigue	–	–	1
pain	–	–	3
sympathy for the target of an unmoral behavior	–	–	3
**Sudden disclosure**	4	–	–
**Action tendencies of the observer**			
fight	7	2	2
flight	–	4	13
freeze	–	3	3
**Actor acts “wrong” yet again**	–	12	14
**Actors behavior seen as done on purpose**	–	–	10
**Tension/ arousal**			
towards actor	12	–	–
independent of actor	6	–	–
intensifier of already upset situation	–	5	–
**Ill-omened**			
because of situational aspects	–	8	–
because of prior behavior of the actor	–	16	–
in terms of a tainted actor-observer relation	–	13	–
because of the state of the observer	–	11	–
**Aftermath even today**	–	3	–

*Notes*. Numbers indicate the recollections containing the described category/ subcategory. Dashes indicate that none of the recollections have such a category/ subcategory. The numbers in bracket beneath anger, indignation and contempt show the number of recollections for these emotions.

Anger. In most of the cases, anger was elicited by a person close to the observer. Anger is also a “close” emotion, because all recollections described the actor as being directly affected by the negative outcomes of a situation. A typical cause of anger was a fellow student who did not prepare sufficiently for a joint presentation. For example, in 6 (of 34) recollections such a perceived injustice or distributional conflicts were described. In nearly all of the interviews (30 of 34) the observer was blocked in some way, a finding specific to anger-experiences, and rather untypical for contempt or indignation:

“(…) my boss wanted me to work on weekends and in the evening with no ifs, ands, or buts, even though I had plans with my kids.” (Subject 131)

In two categories that were named most often within these 30 cases (12 recollections), anger was elicited because the social relationship between the actor and another person was blocked or endangered. This could be either due to a blocked relation to another person, or due to blocked relationships regarding the actor himself. Furthermore, in most of the interviews (29 of 34) the observer’s expectations had been disappointed. Moreover, in half (17) of the 34 interviews, the observer perceived a devaluation or threat to the self.

A noticeable feature of the interviewees’ anger was that it often came with many additional feelings. We found a broad landscape of very subtle and fine graded words (altogether 38 appearances in the 34 recollections) for many different emotions that were named by the participants (12 categories for anger), In 12 (of the 34) situations the observer stated that there was the relationship to the actor had been strained prior to the triggering situation. In contrast to indignation and contempt, anger was described as more approach-related as it led to “fight” reactions (going into confrontation) comparatively often (i.e., 7 of 34 recollections), rather than “flight” or “freezing”. In addition, anger was described as accompanied by a certain amount of tension or arousal which was raised by both, the actor (12 recollections) or by third persons beforehand (6 recollections).

Indignation. In contrast to anger, indignation was about equally often elicited by socially distant (27) and close persons (22). Although the observer felt blocked in some of the recollections (14 of 49), this was comparatively less often than for anger. Unlike anger, the observer did not always have to be victim of a wrong behavior himself to feel indignation, we also found some situations when the observer was not affected at all. Nevertheless, the majority of morally wrong behavior did directly affect the observer (35 recollections). However, in 12 recollections, other persons were affected by a morally bad behavior of the actor. One of the participants experienced indignation because of two friends who were trying to seduce a woman, although both were in a relationship. In another recollection, a dispute between father and son was settled in front of a large group of family members and friends, whereas the observer regarded this as an inadequate social behavior. In this way, indignation was often elicited by inappropriate behaviors.

In a large number of recollections (20) the observer explicitly refers to a specific norm, rule, or role model function:

“He betrayed his girlfriend (whom I knew very well) with another woman and thought it was ok because the stranger (approx. 40 years old) ‘needed this’ and was ‘thankful’. The bad thing is that this person in his 44 years was always a role model for me–well, until this incident happened.” (Subject 91)

Like for anger, the participants mentioned a range of additional negative emotions also elicited in the described situations. A special characteristic for indignation was that the target of emotion behaved wrong repeatedly (12 recollections), a category we did not find for anger or contempt. Moreover, in most of the 49 interviews (36), the actor’s behavior was seen as inappropriate, incomprehensible, or unreasonable in a given context.

Furthermore, in 23 of the 49 interviews, the observer reported a prejudice concerning the actor and in most of the interviews (40 of 49 recollections), feelings of indignation had been ill-omened. For example, this could be because an actor had already behaved negatively to the observer shortly before the recollected situation (16). This was typically described as a negatively appraised behavior prior to the actual behavior that led to indignation.

Contempt. Contempt was (like indignation) mainly elicited within distant actor-observer relationships (39 recollections), rather than in close ones (9). This finding was even more extreme than for anger and indignation. Furthermore, in over the half of situations (25) not only the observer was affected by an actor’s wrongdoing, but other persons were involved. Thus, some situations explicitly described a third person as a victim of the morally bad behavior. This person could be both, either a close relative of the observer (in 14 recollections) or a more distant person (11 recollections).

Just like for anger and indignation, a conspicuous feature of contempt is that it was accompanied with additional feelings. The predominant motivation described by the observer had to do with getting out of a situation, rather than looking for a confrontation (13 of 48 recollections):

“(…) because he misbehaved for a long time and treated me badly. That is why I moved out of the house.” (Subject 42)

Like for indignation, the interviewees described a repetition of the same or similar situation by the actor and/or the observer (14 of 48 recollections). Furthermore, in 10 recollections, the participants characterized the morally bad behavior explicitly as intentional. Like for indignation, we found a lot of interviews describing norm-breaking behavior (e.g., breaking personal taboos in 14 recollections). For contempt, we found very subtle descriptions in this vein. For example, in several interviews (16 of 48 recollections) the actor was lacking respect towards the observer or other third parties involved.

#### Discordant moral observer emotions

Analyses revealed that situations in which the participants experienced schadenfreude or sympathy, different kinds of interaction partners were involved. For both discordant emotions, all reported situations included interactions in which participants were ‘observers’ who witnessed another person (the ‘target of the emotion’) suffering a misfortune.

Examining the interactions eliciting sympathy besides the observer and the target of the emotion, sometimes two other groups of people were involved. The first group were persons responsible for the misfortune of the target of the emotion (present in 13 of 33 recollections). For example, this could be someone who played a trick on the observed person. In what follows, we labeled these persons as ‘wrongdoers’. The second group were persons more or less passively present in the situation (19 of 33 recollections); we labeled these as ‘bystanders’. Therefore, analyzing sympathy, in total four groups of persons were described in the situations.

In comparison, in situations where schadenfreude was elicited, apart from the observer and the target of the emotion ‘bystanders’ were frequently involved (23 of 29 recollections). Thus examining schadenfreude, overall three kinds of persons were described in the situations. Table 5 shows the inductive coding for the two discordant emotions (schadenfreude and sympathy).

**Table 5:** System of Categories and their Frequencies for Schadenfreude and Sympathy.

Category	Schadenfreude	Sympathy
subcategory	(N = 29)	(N = 33)
**Involved in the situation**		
observer	29	33
target of emotion	29	33
wrongdoer	–	13
bystander	23	19
**Closeness of relation from observer to the target of emotion**		
close	11	20
distant	15	4
unfamiliar	3	9
**Damage affected**		
target of emotion	29	23
bystander	2	–
**Observer’s evaluation of target of emotion**		
negative	24	2
positive	1	–
self-comparison	9	–
**Observer’s negative evaluation of the wrongdoer**	–	5
**Target of emotion acts negative beforehand**		
towards observer	12	–
towards bystander	13	–
**Behavior of observer within the situation**		
positive	–	2
negative	2	–
intention	–	4
**Behavior of bystander within the situation**		
positive	–	–
negative	–	12
**Further experienced feelings of the observer**		
respect	–	1
consternation	–	1
helplessness	–	1
guilt	–	1
sadness	–	1
hatred	–	2
contempt	–	1
incomprehension	–	1
anger	1	–
rage	1	–
embarrassment	1	–
sympathy	1	–
satisfaction	1	–
**Experienced characteristics of the situation by the observer**		
controllability of events	–	18
comprehensible/ similar situation	–	12
vice versa-situation	7	–
fulfilling the observer’s predictions	9	–

*Notes*. Numbers indicate the recollections containing the described category/ subcategory. Dashes indicate that none of the recollections have such a category/ subcategory. The numbers in bracket beneath schadenfreude and sympathy show the number of recollections for these emotions.

Schadenfreude. The target of schadenfreude was rather distantly related to the observer, as is the case for acquaintances or competitors (15 of 29 recollections). Observers often explicitly mentioned that targets of schadenfreude suffered due to the misfortune. In contrast, the consequences for the bystanders were rarely mentioned (2 of 29 recollections). The target of schadenfreude who experienced a misfortune was sometimes described as a victim and at the same time as a wrongdoer (sometimes due to wrong attitudes, sometimes due to wrong behaviors). For example, in 24 (of 29) recollections the target of schadenfreude was evaluated as being arrogant or dishonest:

“The other person behaved badly and egotistically towards others and presented herself as being better than she was. But that one time, when it was her turn, she failed miserably. This did not fit the self-image that she communicated to others.” (Subject 35)

Furthermore, in about half of the recollections, the behavior of the target of the emotion had previously been perceived as negative, both towards the observer (12 of 29 recollections) as well as towards bystanders (13 of 29). In contrast, there are rarely descriptions of the observer’s behavior. In two recollections, the observer reported that s/he laughed at the misfortune of the target of emotion. Furthermore, some observers described mixed feelings, i.e., schadenfreude combined with anger, rage, embarrassment, sympathy or satisfaction towards the target of emotion (in 5 of 29 recollections).

In one third of the recollections, there was a self-comparison between the observer and the target of the emotion. In addition, observers sometimes (in 7 of 29 recollections) described a situation when something happened to the interacting persons that he or she was criticized beforehand, and that now happened vice versa. In some cases (9 of 29), the observer was convinced that the misfortune had been predictable, thus, the target of emotion could have prevented the misfortune.

Sympathy. The target of sympathy was typically closely related to the observer, as is the case for family members and friends (20 of 33 recollections). In addition to the observer and the target of sympathy 13 of the 33 recollections included a wrongdoer who was responsible for the misfortune of the target of emotion. In the example below, in addition to the old man being the target of sympathy, a bus driver was described as wrongdoer:

“The trolley drove away just as I approached it and I had to wait 20 more minutes. (…) an old man on crutches appeared, half running (he could hardly walk), as the bus driver simply drove away. Since I knew what it is like to wait for 20 minutes, I felt even worse for him.” (Subject 21)

Furthermore, about approximately two-thirds of all recollections included bystanders (19 of 33 recollections). Observers often explicitly mentioned the emotional and physical suffering of the targets of sympathy; this is the case in two-thirds of the recollections (23 of 33 recollections). In comparison, observers rarely described their own (helping) behavior (2 of 33 recollections). In some cases, the observer had the intention to help, but was not able to (4 of 33 recollections). The inability to help, apart from listening and empathizing, was perceived as burdensome. The behavior of the bystander, if it had been mentioned, was regarded as being negative (12 of 33 recollections). For instance, when the bystanders showed no prosocial behavior towards a helpless person.

Some observers experienced feelings of sympathy in combination with respect, consternation, helplessness, guilt or sadness towards the target of emotion (5 of 33 recollections), and feelings of hatred, contempt, and incomprehension towards the wrongdoer (4 of 33 recollections). In approximately half of the cases (18 of 33 recollections) the participants perceived the situation as being uncontrollable for the target of the emotion. In addition, observers sometimes pointed out that they experienced sympathy because they empathized with the target of emotion and his/her situation (12 of 33 recollections). The consequences of a misfortune were particularly comprehensible when the observer had experienced a similar situation.

## Discussion

In this paper we analyzed 312 recollections of interpersonal events involving either positive moral emotions (respect, admiration, pride, and sympathy) or negative moral emotions (anger, indignation, contempt, and schadenfreude). The variety and intricacy of events recalled by our respondents is impressive: On the one hand, interpersonal events eliciting moral emotions are frequent and commonplace phenomena, and typically involve ordinary and common incidents as they happen to all of us day by day. On the other hand, it becomes apparent from each of the recollections we sampled that these emotions move us deeply and are indeed highly ‘emotional’ experiences. Thus, we are well-advised to take these feelings serious, as they have a deep impact on the way we perceive, feel and act.

Our analyses of these autobiographical recollections so far have been based on two strategies: First, we have been guided by theoretical concepts—originally proposed by Heider [2], and recently taken up by Weiner [4] and Rudolph et al. [3]. Second, our approach is characterized by qualitative analyses allowing for a deductive test as to whether our theoretical concepts are present in naïve accounts of everyday situations (deductive approach). In addition, we were able to discover specific features of otherwise similar emotions by inductive research strategies. The valence or hedonic quality of moral emotions strongly influences important characteristics of our autobiographical recollections: Negative moral emotions (and thus, negative events) elicit more extensive descriptions of the antecedents and consequences of the respective interpersonal events (as compared to positive moral emotions and positive events). This effect is especially strong in case of pride on the positive side, and contempt on the negative side. There are several explanations for this effect. It might be that negative events are processed more deeply, and thus remembered better [50]. In addition, the density hypothesis [51] implies that positive information is processed faster because of a higher homogeneity of positive events as compared to negative ones. Finally, in line with this reasoning, attributional findings (e.g., [52]) suggest that surprising, personally important, and negative events tend to elicit causal analyses. As a consequence, negative events and emotions should cause a more elaborated attributional search, thus resulting in more extensive descriptions.

### Ought, Goal-attainment, and Effort as Determinants of Moral Emotions

We have seen that ought, goal-attainment and effort are consistently present in our recollections of autobiographical events. Moreover, the most frequent configurations of these concepts (as mentioned by our participants) fit perfectly well to those patterns that have been derived from our theoretical considerations. Although values vary slightly between the different emotions, 65% and 100% of recollections are characterized by the theoretically predicted patterns. Given the enormous variety of emotional experiences described by our participants, this is a strong indicator of the predictive power of these variables. Hence, previous theoretical considerations and thought experiments [1–3,7] are now corroborated on new methodological grounds. It is also evident that members of the respective clusters of moral emotions–that is, positive, negative, and discordant emotions–are based on similar configurations of ought, goal-attainment and effort, respectively. On a theoretical level, we replicated previous findings [3] indicating that the concepts ought, goal-attainment, and effort strongly predict moral observer-emotions. With astounding regularity, the postulated conceptual patterns predict specific emotions or sub-groups of emotions. For example, the vast majority of recollections of positive moral emotions described situations in which the observed person had expended high effort to attain a morally positive goal that is eventually attained. In contrast, negative moral emotions typically arise in situations when the observed person pursues a morally negative goal.

From a functionalist perspective, we now see that positive moral observer emotions like pride, admiration, and respect signal that another person’s behavior has been morally right, which is most likely given the combination of morally positive goals that are attained under high effort conditions. In addition, we also learn that among the three emotions, the presence of effort is most important for pride.

In contrast, negative moral emotions like anger, indignation, and contempt signal that another person’s behavior has been morally wrong. This is most likely when morally negative goals that are attained. Moreover, it becomes apparent that effort plays a crucial role in interactions eliciting anger and indignation, as these emotions also arise when positive goals are not attained due to a lack of effort. Finally, our data hint at an especially important difference between positive versus negative moral observer emotions: Positive moral emotions are elicited when moral standards are met or exceeded (see also, [9]), while negative moral emotions most often occur following a morally negative behavior or a lack of positive behaviors such as lack of effort [4] (e.g. transgressions or violations of moral standards; see also, [23]).

We also provided a closer analysis of the so-called discordant emotions. It turns out that sympathy and schadenfreude occur when a positive goal is not attained. In contrast to sympathy, schadenfreude may also arise in situations when a negative goal is not attained [53,54]. While non-attainment of a goal is an inevitable prerequisite to experience either sympathy or schadenfreude, effort plays a minor role for the discordant emotions. This finding clearly distinguishes the discordant moral emotions from the remaining moral observer emotions.

Finally, the functional value of the discordant emotions is complex, as functional value and hedonic quality are inverted: For sympathy, which feels bad, we have a positive signal conveying that the respective behavior is morally right. The autobiographical recollections of our participants typically consist of descriptions of positive goals that had been pursued with high effort. In contrast, schadenfreude feels good–however, this emotion signals that another person’s behavior has been wrong. In line with these considerations, the autobiographical recollections of our participants typically depict morally negative goals that have been pursued (see also, [1]).

In sum, our quantitative analyses suggest that Heider’s naïve action analysis and thus the concepts of ought, goal-attainment, and effort predict a large variety of moral emotions. We also believe that our methodological approach is especially useful here: By using the described conceptual analyses based on the autobiographical recollections generated by our participants, the information given by our participants is unbiased by demand characteristics of the investigation.

### Beyond A-Priori Concepts

Let us now turn to our inductive qualitative analyses and the insights we gain from these. We will do so by separately analyzing each group of moral observer emotions. Here, we will also refer to conditions of moral emotions as already addressed by previous research. Note that our summary of our participant’s recollections will predominantly concentrate on those findings that (1) were reported most frequently by our participants or (2) have not been mentioned by previous approaches. For more detailed information, please refer to Tables 3–5.

#### Positive moral emotions

The present analyses suggest that in addition to ought, goal-attainment, and effort, high abilities, exceptional talents and positive personality traits play an important role in the context of positive moral emotions. This observation is in line with previous analyses [20,29]. Some interesting differences between these three emotions become apparent as well: First, pride and respect are more often experienced in achievement contexts. It is evident that effort plays a crucial role in this context [5,6], and events characterized by high effort elicit especially strong feelings of pride and respect. In contrast, admiration is an emotion more closely related to cases in which we highly esteem another person’s outstanding personality or ability.

To take up considerations by Tangney and coworkers [20,29], these results extend concept of alpha- (pride related to the self, e.g., abilities) versus beta-pride (pride related to behaviors) to observer emotions: We now see the similarities and differences of pride on hand and admiration and respect on the other hand. All three positive moral emotions can be elicited by observing positive traits or abilities. However, the present data reveal that admiration and respect (in contrast to pride) are typically related to achievements or abilities the observer does not have but would like to have. Finally, pride is typically experienced in close relationships (see also, [10]), especially when the observer is involved in the reported situation. In contrast, admiration and respect are more frequently experienced with regard to more distant persons.

#### Negative moral emotions

Indignation and contempt are predominant in situations with distant interaction partners, whereas anger is elicited mainly in close relationships. This is in line with earlier findings [55], emphasizing a stronger social exclusion function of contempt and indignation as compared to anger [23]. Rather, anger is elicited when expectations related to the interaction partner are not met, or when personal goals are blocked. As personal goals are often connected with close relatives, anger motivates to immediately change another person’s behavior [56]. Anger therefore can be regarded as an approach-related emotion. Contrary to anger, indignation and contempt are especially strong when a morally wrong behavior is observed repeatedly. For indignation, it is often the case that a certain code of conduct is transgressed, whereas contempt is typically elicited vis-à-vis bad characteristics or traits of another person. Hence, the behavioral consequences of anger (confrontative, fighting) as compared to indignation and contempt (social exclusion) are entirely different [56].

#### Discordant emotions

For schadenfreude, relationship between observer and target of the emotion is typically negative, for example due to undesirable behaviors of the interaction partner prior to the misfortune. Such undesirable behavior obviously encourages dislike and resentment in the observer (see also, [15]). The resulting negative attitude of the observer may lead to a variety of feelings, such as anger, rage, embarrassment or satisfaction. The recollections of our participants reveal that the pleasure in schadenfreude is due to the fact that the observer engages in a self-other comparison (with the target of schadenfreude as a comparison object). This is in line with our observation that observers experiencing schadenfreude typically recognize that the target of schadenfreude suffers due to the misfortune. Hence, a situation involving schadenfreude often seems to provide an opportunity for a more favorable self-view and self enhancement [57].

Moreover, persons experiencing schadenfreude typically attempt to hide the emotion, and are aware of the fact that the joy one takes in the misfortune of another person is hurtful and potentially maladaptive in social situations [24,58]. Accordingly, our participants only rarely report high levels of schadenfreude. Schadenfreude typically appears when a person is convinced that the misfortune has been predictable and thus preventable. For example, schadenfreude is especially likely when an interaction partner suffers a misfortune because s/he disregarded the advice of the person who subsequently experiences schadenfreude. Actually, it is often the case that the target of schadenfreude could have prevented the misfortune (nota bene, according to the point of view provided by the person experiencing schadenfreude). One might say that the misfortune is deserved in such cases, which is well in line with previous considerations [2,53,54,59].

Recollections of sympathy typically involve close relationships. Furthermore, sympathy often involves a deeper understanding of how difficult a situation is for another person. It is noteworthy that sympathy is triggered by both emotional and physical signs of suffering, as we know that these signs of suffering strongly predict prosocial actions [1]. Finally, the present data clearly confirm attributional accounts of sympathy [4]: We observe that the misfortunes that happened to the target of sympathy are unequivocally perceived as being uncontrollable, which, in turn, predicts help-giving [26].

### Limitations and Implications for Future Research

We analyzed incidents of moral emotions in autobiographical recollections. It turns out that these recollections involve an astounding variety of everyday-events. At the same time it becomes clear that these everyday events are regarded as important and severe, and the emotional character of these situations contributes strongly to the fact that these situations are taken seriously by our participants. However, note that our methodological approach cannot give precise information about the frequency and the strength of these emotions and their antecedent conditions.

Thus, an important task for future research is to analyze the frequency and strength of emotional events and their antecedent conditions. One research strategy in this vein might include to further elaborate on the situations provided by our participants, as these recollections provide sufficient richness and variety which is difficult to achieve when relying solely on the creativity of the researchers (which have to artificially create or ‘invent’ suitable scenarios).

Finally, one specific conclusion to be drawn from the present data is that it is only rarely the case that one set of eliciting conditions exclusively tied to one specific moral emotion. Rather, such preconditions provide (varying) probabilities with which these elicit subsequent emotions, and there is considerable overlap between various sets of preconditions and groups of emotions. Such variety may have different origins: On one hand, this speaks to the richness of our emotional experiences. On the other hand, it is also clear that we still need to explore the individual differences in experiencing and expressing these moral emotions in different settings.

## Appendix A

Quotes for the Categories Found for Respect, Admiration, and Pride

### Pride

This emotion was experienced when the observer is involved her/himself (21 of 36 recollections):

“When my little niece painted her first picture on her own. Previously I have helped her, e. g. showed her how to paint a sun. (…) she has always asked ‘can you help me’, until she was able to draw her own picture without asking me for help.” (Subject 43)

The fact that pride was often elicited in contexts referring to personality development (17 of 36 recollections) is apparent in the following example:

“My sister did not feel like going to school when she was young. That is why she attended school only sparsely, and sometimes she totally refrained from going there. In the end she did not finish high-school even though she was quite smart. For some years she was able to stay afloat with a lot of side jobs or by commencing training. She was never satisfied with that and one day decided to make up lost time. She started and graduated secondary school and even high-school!” (Subject 69)

### Admiration

This emotion was directed at personality and/or ability characteristics of the admired person:

“She danced very beautifully, moved very gracefully and womanly and especially with a lot of expression. The movements were very fine-tuned, and the overall picture fit just perfectly.” (Subject 44)

Admiration is elicited by sacrifices to achieve a higher goal (13 of 39 recollections):

“A friend of mine became unexpectedly pregnant at 20. I salute her for what she shouldered in this hard time, and the way she copes with her life which of course has gone a very different direction from what she imagined a few months ago. She works hard for her child and acts very mature. I don’t think everyone could do that and I think many people would fail at such a task, which deserves my admiration.” (Subject 48)

### Respect

This emotion was most often accompanied by being impressed by the observed person (9 of 44 recollections):

“My friend received positive feedback concerning his examination, I now hold him in high esteem because he obtained these good results due to hard work and discipline.” (Subject 81)

The following quote shows, how respect is related to altruism and help-giving (12 of 44 recollections):

“Although the two climbers described in the documentary knew that nobody had climbed the mountain before, they nevertheless attempted to climb the mountain. What really touched me was that the climbers went back to help their rivals, who, because they had started a day later, could not continue due to poor weather conditions. All of the mountaineers had the goal to climb the mountain. Furthermore, it was also about the title: who climbed the mountain first. Although the first two climbers were very close to their destination, they returned to help their rivals.” (Subject 73)

In addition, respect often occurred with regard to abilities or competencies the observer was not able to show or not have her/himself (15 of 44 recollections):

“(…) I came to know a fellow student who was just 19 years of age and traveled to India for a whole year. She learned about the country and its people and even worked with handicapped children! She had such courage and self-confidence, being a million miles away from home, inside a completely new culture doing things that fascinated me. (…) Doing what she did, I would have been under enormous stress even in familiar locations. (…) I had respect for her maturity because I wouldn’t have been capable to live like this as a 19-year-old!” (Subject 73)

## Appendix B

Quotes for the Categories Found for Anger, Indignation, and Contempt

### Anger

In 6 (of 34) recollections a perceived injustice or distributional conflicts were described for the emotion of anger:

“So this person relaxed in the evening while others had to make a real effort once again, still the grades were the same for all.” (Subject 36)

In 12 recollections, anger was elicited because the social relationship between the actor and another person was blocked or endangered. This could be either due to a blocked relation to another person, or due to blocked relationships regarding the actor himself:

“It has come to this situation with these people because they did not want to accept that I had a relationship with my girlfriend.” (Subject 27)

Furthermore, in most of the interviews (29 of 34) the observer’s expectations had been disappointed:

“I was so furious and angry because I never thought that a friend like her could betray me or would blame me for alleged incorrect and mean behavior.” (Subject 14)

Moreover, in half (17) of the 34 interviews, the observer perceived a devaluation or threat to the self, as this quote reveals:

“I apologized and felt very ashamed. I even asserted not to make a mistake like this again and to change my behavior (…) then both of the girls began to smile (…) and I stood there, felt alone, and betrayed. I was so mad about both of them.” (Subject 3)

A noticeable feature of the interviewees’ anger was that it often came with many additional feelings. We found a broad landscape of very subtle and fine graded words (altogether 38 appearances in the 34 recollections) for many different emotions that were named by the participants (12 categories for anger). For example:

“The situation was pretty embarrassing for me because this reaction was totally inappropriate and it annoyed me very much.” (Subject 111)

In 12 (of the 34) situations the observer stated that there was the relationship to the actor had been strained prior to the triggering situation:

“Generally one can say that from time to time there are conflicts and frictions between us siblings because of the daily household tasks (to wash up, tidy up, etc.).” (Subject 44)

### Indignation

Unlike anger, the observer did not always have to be victim of a wrong behavior himself to feel indignation, we also found some situations when the observer was not affected at all. Nevertheless, the majority of morally wrong behavior did directly affect the observer (35 recollections):

“Anyhow, the eBay seller was deleted online and we never caught sight of the laptop and our money was gone.” (Subject 5).

However, in 12 recollections, other persons were affected by a morally bad behavior of the actor:

“A girl raised a question and in response, a very irritable girl said: ‘Well, if you do not even know that, then you do not belong here (…) how did you graduate from high-school?’” (Subject 76).

Moreover, in most of the 49 interviews (36), the actor’s behavior was seen as inappropriate, incomprehensible, or unreasonable in a given context:

“(…) she showed no responsibility at all (…). Obviously, she has a completely different definition of the situation as she wants to send her own mother into an asylum.” (Subject 12)

“Just for no reason, not knowing the slightest bit about a person, to attack him and a little child in my opinion is simply malicious.” (Subject 81)

In 23 of the 49 interviews, the observer reported a prejudice concerning the actor:

“The patient emphasized over and over again that I am still very young and did not have a lot of experience (…). There was a friendly and polite interaction but always with a certain overtone.” (Subject 25)

In most of the interviews (40 of 49 recollections), feelings of indignation had been ill-omened. This was typically described as a negatively appraised behavior prior to the actual behavior that led to indignation:

“Even before that the girl made her presence felt by continually pushing us from behind and exclaiming, ‘hurry up! I need to be first, I'm in a hurry!’ Something like that is just selfish”. (Subject 30)

### Contempt

In over the half of situations (25) not only the observer was affected by an actor’s wrongdoing, but other persons were involved.

“I felt contempt for my father’s new partner as she always drove to my mother’s house after my parent’s divorce again and again. There she complained about my father and she plagued my mother with the details of their partnership, although the divorce was very hard for my mother.” (Subject 44)

A conspicuous feature of contempt is that it was accompanied with additional feelings:

“I was extremely disappointed and angry and in some way I despised her because of that.” (Subject 29)

Like for indignation, the interviewees described a repetition of the same or similar situation by the actor and/or the observer (14 of 48 recollections):

“Furthermore that was not the first time that my father yelled at anyone. In my family he is therefore despised and that bothers me.” (Subject 50)

Furthermore, in 10 recollections, the participants characterized the morally bad behavior explicitly as intentional:

“(…) Although he realized that I was feeling very bad and I was like leaving out crying, he continued making a fool of me!” (Subject 66)

For contempt, we found very subtle descriptions for norm-breaking behavior. For example, in several interviews (16 of 48 recollections) the actor was lacking respect towards the observer or other third parties involved:

“And as he expects that I respect him, I fondly hope to get the same from him. However, in this situation he wanted to demonstrate his power and to show who has more pull.” (Subject 12)

## Appendix C

Quotes for the Categories Found for Schadenfreude and Sympathy

### Schadenfreude

The target of schadenfreude was rather distantly related to the observer, as is the case for acquaintances or competitors (15 of 29 recollections):

“When I had been going to school, there was a person who always cheated or copied and never did her own homework. Once she had been caught, it amused me very much.” (Subject 9)

“There was this fellow student of mine who always pretended that everything would be very easy (…). She treated me and others very condescendingly. Five or six weeks ago she screwed up a moderation because she had not prepared herself properly again, but this time she did not get away with it.” (Subject 8)

Observers often explicitly mentioned that targets of schadenfreude suffered due to the misfortune. In contrast, the consequences for the bystanders were rarely mentioned (2 of 29 recollections):

“Once my team and I took part in an indoor football tournament: We faced another team who we would stand no chance against within the normal season (…). For the players of the other team, the competition was won from the outset. Indeed, the game was balanced and in the end we won it. (…) Then he (a member of the opponent team) started to play unfairly and became offending towards the other players and the referee. I felt like gloating after he was punished for his arrogance and his attitude/behavior that affected his team.” (Subject 91)

Furthermore, in about half of the recollections, the behavior of the target of the emotion had previously been perceived as negative, both towards the observer (12 of 29 recollections) as well as towards bystanders (13 of 29):

“My girlfriend is actually very lovely, but is also often very snobbish. She asked me for a grammatical suggestion, and as I am interested in this matter, I wanted to explain it well. But she only laughed at me and teased me again and again since then. Recently she has done this in front of the whole group; this was very embarrassing. But then many others were quite uninterested and said that this was quite clear and that there was nothing to laugh about.” (Subject 10)

In one third of the recollections, there was a self-comparison between the observer and the target of the emotion:

“Schadenfreude has been triggered when a seemingly arrogant person stumbled while she was staring at me disapprovingly (…). The person was unknown to me, but was about the same age and the same sex as me.” (Subject 29)

In addition, observers sometimes (in 7 of 29 recollections) described a situation when something happened to the interacting persons that he or she was criticized beforehand, and that now happened vice versa:

“My man locked himself out of the apartment late at night, wearing just underpants. Recently the same thing happened to me, and (he) was surprised that "such a thing" could ever happen.” (Subject 101)

In some cases (9 of 29), the observer was convinced that the misfortune had been predictable, thus, the target of emotion could have prevented the misfortune:

“When my ex-boyfriend had a sprained ankle (…) I told him to rest. He always knew best, no matter what. He was always like ‘Yes, but…’ Even when it comes to medical stuff, he says that I would be ‘only a nurse’. Then he went jogging and it happened… the joint became completely unstable and he rolled his ankle suffering a massive ligament injury. I did not feel bad for him and honestly I even laughed and I thought that it served him right.” (Subject 11)

### Sympathy

The target of sympathy was typically closely related to the observer, as is the case for family members and friends (20 of 33 recollections):

“My friend was cheated on by her boyfriend after the two were together for two years (…). She felt very depressed and was barely approachable. She is usually a very happy person, but that time she did not laugh for days and that is why I felt much more compassion for her.” (Subject 17)

Furthermore, about approximately two-thirds of all recollections included bystanders (19 of 33 recollections):

“In a lottery a winner was determined who was to come on the stage, the winner stuttered very strong, there was laughter in the hall” (Subject 15)

Observers often explicitly mentioned the emotional and physical suffering of the targets of sympathy (23 of 33 recollections):

“Suddenly the mother tugged the girl’s earlobe, twisted it (the lobe), and yelled at her, resulting in that little girl crying. And right after that, the same thing happened to the boy. The mother twisted and tugged the little boy’s lobe and shouted at him (…). To witness how the children suffered from pain caused us to feel deep compassion.” (Subject 41)

The behavior of the bystander, if it had been mentioned, was regarded as being negative (12 of 33 recollections). For instance, when the bystanders showed no prosocial behavior towards a helpless person:

“A woman wanted to get on the bus (…) and was pushed by some passersby, so that she fell. The woman was disabled and used walkers. She helplessly laid on the ground for a long time until a man helped her up. (…) Many people just stared at her and watched her squirming. (Subject 7)

Furthermore, some observers experienced feelings of hatred, contempt, and incomprehension towards the wrongdoer (4 of 33 recollections).

In approximately half of the cases (18 of 33 recollections) the participants perceived the situation as being uncontrollable for the target of the emotion:

“I felt sympathy for my cousin (…) who has had a serious disease and now sits in a wheelchair. The disease is incurable and it is likely that he dies before I do. This makes me very sad and I feel sympathetic towards him. It is just the situation that he is in a wheelchair and cannot do anything about his disease.” (Subject 38)

In addition, observers sometimes pointed out that they experienced sympathy because they empathized with the target of emotion and his/her situation (12 of 33 recollections). The consequences of a misfortune were particularly comprehensible when the observer had experienced a similar situation:

“Recently I talked to a friend of mine who just finished high-school (…) it appeared that she had not been accepted for a job although she applied often. (…) I feared about my favorite course of study and could comprehend how she had to feel.” (Subject 28)
